# Supplementary material for: Continuous Monitoring of Vital Signs Using Cameras: A Systematic Review
Source: Sensors (Basel). 2022 May 28;22(11):4097. doi: 10.3390/s22114097 (PMC9185528; doi:10.3390/s22114097)
Supplement: Supplementary file 1 [file sensors-22-04097-s001.zip › sensors-1681808-supplementary.pdf]

sensors

MDPI

Table S1. Extracted information from all articles

No	Article	Vital sign	Database	No. of subjects	Male	Female	Age (years)	Participant type	Skin tone	Settings	Camera	Camera details	Resolution	Frame rate (fps)	Illumination	Distance	Ground truth	Protocol
1	[12]	SpO_2	Self-record	5	4	1	25-38	Healthy	Fit scale II, III, IV	Indoor	RGB	iPhone X	-	60	Phone flash light	30 cm	Pulse oximeter, blood pressure cuff	Sitting comfortably and quietly
2	[20]	HR	Self-record	20	-	-	23-26	Healthy	Light, Brown medium	Indoor	RGB	Webcam	720p	-	Illumination constant	1-3 m	Ambiotex smart shirt, Fitbit Alta HR	Stay relax
3	[22]	HR	Self-record	12	12	0	-	-	-	Indoor	RGB	Logitech C270	640X360	30	-	1 m	ECG	Stay still, facial expressions
			MAHNOB-HCI	30	13	17	26.36 +/- 4.39	-	-	Indoor	RGB	Allied Vision Stingray F-046C	780X580	60	-	40 cm	ECG	Facial expression and voluntary head motion
4	[24]	RR	Self-record	17	11	6	5.3 +/- 3.1 weeks	Preterm infants	Indian, Southeast Asian	Indoor	YUV	IP camera	320X180	10	Various lighting	4-6 feet	ECG impedance pneumography	Infants lying in incubator bed
5	[25]	RR	Self-record	7	5	2	1-59 days	Infants	-	Indoor	LWIR	FLIR Lepton 2.5, FLIR lepton 3.5	60X80, 120X160	8.7	-	-	Philips MX700	Infant sleeping in open bed
6	[26]	RR	Self-record	15	-	-	1-77 days	Infants	-	Indoor	RGB, LWIR	IDS UI-2220SE; FLIR Lepton 2.5	576*768; 60*80	20, 8.7	-	-	Philips patient monitor MX800	Infants lying in bed
7	[27]	RR	Self-record	12 adult, 8 newborn	7 adult, 6 newborn	5 adult, 2 newborn	21-31 adult, Newborn: 27 +/- 19 days	Healthy	-	Indoor	LWIR	VarioCAM HD head 820 S/30 mm (InfraTec GmbH)	1024X768	30	-	2 m	piezo plethysmography, surface ECG	Adult: sit normally, simulated breathing, Infant: lying in bed
8	[29]	RR	Self-record	27	18	9	48.6-74.3	ICU patients	-	Indoor	LWIR	Optrics PI 450	382X288	80		> 1 m, 0.4-0.6 m	Manual counting	Lying in supine position
9	[31]	RR	Self-record	20	19	1	68.9± 11.2	Anesthesia Patients	-	Indoor	Thermal	FLIR T-420	320X240	10	-	50 cm	BP, HR, Pulse oximeter, Respiratory volume monitor	Lying in bed and observed before and after midazolam dosage
10	[32]	RR	Self-record	16	6	10	25-37	Healthy	-	Indoor	LWIR	Seek Thermal Compact PRO for iPhone	640X480	17	No light	100 cm	Go direct respirator belt	Sleeping
11	[36]	HR	Self-record	In lab: 12; In-car: 1	9	3	20-40	Healthy	varying	Indoor and outdoor	RGB, NIR	FLIR Backfly BFLY-U3-23S6C-C, Point Grey Grasshopper GS3-U3-41C6NIR-C	640X640	30	NIR light and ambient light	-	Pulse oximeter	In-lab: Sit still, but to allow for natural head motion; In-car: passenger in car
12	[37]	BP	Self-record	5	5	0	27.0± 9.5	Healthy	-	Indoor	RGB	Imaging source DFK33UX252	640X480	60	White LED (800 lux)	33 cm	Sphygmomanometer	Pedaling exercise
13	[39]	HR	Self-record	24	13	11	23-38	Healthy	Western European	Indoor	RGB	Logitech c920	1280*720	30	Ambient, screen light	60 cm	ECG	Rest, stress test

**14**	[40]	HRV	Self-record	136	57	79	24.33 ±8.62	Healthy	-	In-door	RGB	Canon VIXIA HF R62	-	60	LED light	60-90 cm	ECG	Sitting naturally and watching cloud video
**15**	[43]	HR, RR	Self-record	25	15	10	20-30	Healthy	Fitz scale I-VI	In-door	RGB	Smartphone LG G2	-	30	Flash light	~0.5 m	HR: pulse oxi-meter, RR: Manual in-spection	Stay still with minimal move-ments
**16**	[49]	HRV	Self-record	50	39	11	Mean 27	Healthy	-	In-door	RGB	iPhone 6	-	240	Flash light	-	ECG	Sit relaxed position
**17**	[51]	HR, RR	Self-record	12	8	4	23-34	Healthy	Asians, Africans and Cau-casians	In-door	NIR	Monochromatic infrared camera	640X240	62	Infrared LED	varied	Pulse oxime-ter, respiratory belt	Seated at a desk naturally and breathe spontaneously at light and dark room
**18**	[52]	RR	Sleeping dataset,	12	10	2	21-38	-	-	In-door	NIR, ther-mal	Thermal imager MAG62	640X480	-	Dark environ-ment	1-3 m	Manual	Sleeping
			Self-record	11	-	-	-	-	-		NIR	Avigilon H4 HD Dome	-	-		2.87-3 m	Manual count using counter app	Sleeping
**19**	[53]	HR, RR	Self-record	50	30	20	18-85	Subjects with hy-perten-sion, my-asthenia gravis, and dia-betes	-	In-door	NIR	Point Grey Fire-fly MV	640X480	30	Infrared light	1.5 m	Polysomnog-raphy, ECG, Inductance plethysmogra-phy	Sleeping
**20**	[55]	HR	Self-record	20	17	3	20's-60's	-	-	In-door	RGB, NIR	dual CCD RGB-NIR camera AD-130GE	1296X964	30	Fluorescent light, NIR LED light for NIR wave-lengths, com-puter screen illumination.	1.5 m	Contact PPG sensor	Sit still in the chair and watching movie
**21**	[56]	RR	Self-record	6	-	-	-	-	-	In-door	RGB	Canon camera	-	-	Well-lit illumi-nation		PPG sensor, Respiratory belt	Sit rest with motions and expres-sions
**22**	[57]	HR, RR	Self-record	20	13	7	27.85 ±6.9	Healthy	-	In-door	MWIR	Infratec Im-ageIR 9300	1024X768	50	-	1.5 m	piezoplethys-mography, In-telliVue MP70 patient moni-tor	Sit still, sit side 90 degree and still as possible
**23**	[59]	HR	Self-record,	15	13	2	22-25	-	Asian	In-door	RGB	GoProHERO6 Black	2704X1520	30	Natural sun-light	0.5, 1, 1.5, 2, 2.5, 3.0 m	ECG	Sit stationary
			UBFC-rPPG	42	-	-	-	-	-	In-door	RGB	Logitech C920HD pro	640X480	30	Sunlight with ambient illu-mination	1 m	Pulse oximeter finger clip sen-sot	Sitting with Rigid and non-rigid motion, mathematical game
**24**	[60]	RR	Self-record	30	13	17	27±3	Healthy	Fitz scale: I-V	In-door	NIR, FIR	NIR: see3cam_CU40, FIR: FLIR lep-ton version 3.5	NIR: 336X190 pixels, FIR: 160X120 pixels	NIR: 15, FIR: 8.7	NIR LED ar-ray	-	Piezo resistive based respira-tor belt	Lay in bed in supine position and breath through nose at dark room

**25**	[61]	BST	Self-record	596	IRT1: 215, IRT-2: 212	IRT1: 329, IRT-2: 328	18+	Healthy	white, Black/african-american, Hispanic/Latino, Asian, Multiracial, american Indian	Indoor	RGB, IRT	RGB: Logitech webcam C920, IR-1: FLIR A325sc, IR-2: FLIR 8640 P-series	RGB: NA; IRT-1: 320*240, IRT-2:640*512 pixels	30	Ambient light	0.6-0.8 m	Oral thermometer	Sit Infront of the camera
**26**	[75]	HR	MMSE-HR	40	17	23	18-66	-	-	Indoor	RGB	Di3D dynamic imaging system	1040X1392	25	-	-	ECG	Emotion variance
**27**	[76]	HR	MAHNOB-HCI	27	12	15	19-40	-	-	Indoor	RGB	Allied Vision Sting-ray F-046C	780X580	61	-	40 cm	ECG	Emotion elicitation
**28**	[77]	HR	MAHNOB-HCI	27	12	15	19-40	-	-	Indoor	RGB	Allied Vision Sting-ray F-046C	780X580	60	-	40 cm	ECG	Emotion elicitation and implicit tagging data
			Self-record	40	28	12	18-60	-	-	Indoor	RGB	Logitech C920	640X480	30	Ambient light	50 cm	ECG	Static – motionless and expressionless, dynamic – turns head and changed the expressions
**29**	[78]	HR	PFF	13	-	-	-	Healthy	-	Indoor	RGB	Nikon D5300	1280X720	50	Fluorescent lamp	0.5 m	Two Mio Alpha II wrist HR monitor	Sit still with lights on and off, movements with lights on and off, riding exercise bike with lights on
			PURE	10	8	2	-	-	-	Indoor	RGB	evo274CVGE	640X480	30	Daylight through window	-	Contact PPG sensor	stay still, talking, slow translation, fast translation, small rotation, medium rotation,
			MAHNOB-HCI	27	12	15	19-40	-	-	Indoor	RGB	Allied Vision Sting-ray F-046C	780X580	60	-	40 cm	ECG	Facial expression and voluntary head motion
			VIPL-HR	107	79	28	22-41	-	-	Indoor	RGB, NIR	1) Logitech C310, 2) RealSense F200, 3) HUAWEI P9	1)960X420 2)640X480 (NIR); 1920X1080 (RGB) 3) 1920X1080	1)25, 2)30, 3)30	Filament lamp	1 m, 1.5 m	Pulse oximeter	Head movements, illumination variations
**30**	[79]	HR	PURE	10	8	2	-	-	-	Indoor	RGB	Evo274CVGE	640X480	30	Day light through window	-	Contact PPG sensor	stay still, talking, slow translation, fast translation, small rotation, medium rotation
**31**	[80]	HR	MMSE-HR	40	17	23	18-66	-	-	Indoor	RGB	Di3D dynamic imaging system	1040X1392	25	Asymmetric light	-	ECG, blood pressure - Biopac MP150	Emotional reactions
			PURE	10	8	2	-	-	-	Indoor	RGB	Evo274CVGE	640X480	30	Day light through window	-	Pulse oximeter	stay still, talking, movement
**32**	[81]	HR	Self-record	65	34	31	-	-	-	Indoor	RGB	Logitech webcam C270	640X480	30	-	-	Pulse oximeter	Natural facial movements and head pose variations

			COHFACE	40	28	12	35.6± 11.47	Healthy	-	Indoor	RGB	Logitech C525	640X480	20	Studio lighting & sunlight	-	PPG sensor, Respiratory belt	sit still with varying illumination (studio and natural lighting)
**33**	[82]	HR	MMSE-HR	40	17	23	18-66	-	-	Indoor	RGB	Di3D dynamic imaging system	1040X1392	25	-	-	ECG	Facial expression
**34**	[83]	HR	FAVIP dataset	15	12	3	23-45	-	Varying skin complexion	Indoor	RGB	Samsung galaxy S3, iPhone 3GS	1280X720	30	-	6 inches, 1 foot, 2 feet	Pulse oximeter	Sitting normally with varying distance
**35**	[84]	HR	Self-record	18	-	-	-	-	-	In-door	RGB-NIR	JAI AD-130GE	1296X966	30	NIR flash	-	Pulse oximeter	Sit still
			Tokyo Tech dataset	9	8	1	20s-60s	-	-	In-door	RGB-NIR	RGB-NIR sensor	640X480	30	KINOFLO lights, NIR lights	1.5 m	Contact PPG sensor	Relax, exercise-handgrip exercise, relax
			MR-NIRP dataset	8	6	2	20-40	Healthy	Indian, Caucasian, Asian	In-door	RGB, NIR	Point Grey Flea3 FL3-U3-13E4C-C, Point Grey Grasshopper GS3-U3-41C6NIR-C	640X640	30	Ambient overhead lights	-	Finger pulse oximeter	Sit still and motion task
			UBFC-rPPG	47	-	-	-	-	-	In-door	RGB	Logitech C920 HD pro	640X480	30	Sunlight and indoor illumination	1 m	Pulse oximeter finger clip sensot	Sitting with playing mathematical game
**36**	[85]	PRV	MMSE dataset	140	58	82	18-66	-	Europeans, Middle Easterners, South Asians, South Americans and East Asians	In-door	RGB	3D dynamic imaging system, FLIR A655sc Longwave infrared camera	1040X1392, 640X480	25	Symmetric lights	-	Contact PPG sensor	Sitting with varying facial movements
**37**	[86]	HR	COHFACE	40	28	12	35.6± 11.47	Healthy		In-door	RGB	Logitech HD C525	640X480	20	Well- lit and natural	-	PPG sensor, Respiratory belt	sit still with varying illumination (studio and natural lighting)
			UBFC-rPPG	42	-	-	-	-	-			Logitech C920 HD Pro	640X480	30	Varying indoor light and sunlight	1 m	Pulse oximeter	Mathematical game
			PURE	10	8	2	-	-	-			eco274CVGE	640X480	30	Daylight through window	-	Pulse oximeter	Sit still, Motion, moves the head, rotation of head
**38**	[87]	HR	Self-record	45	-	-	21-63	Healthy	Fair, Brown, Black	In-door	RGB	Bayer mosaic	1080X1920	30	Fluorescent light (340-380 lx, 430-470 lx, 510-550 lx)	80 cm (500, 700, 1000 cm)	Pulse oximeter	Human computer interaction, Health monitoring scenario
			MAHNOB-HCI	27	12	15	19-40	-	-	In-door	RGB	Allied Vision Sting-ray F-046C	780X580	61	-	40 cm	ECG	Emotion elicitations
**39**	[89]	RR	Self-record	20	-	-	-	Healthy	-	In-door	RGB	Logitech C922 Pro / GigE Sony XCG-C30C	1280X720 / 659X494	60/60	-	0.5-1 m	Pulse oximeter, respiratory belt	Sit still with a little movements and constant breathing frequency, spontaneous breathing
**40**	[91]	RR	Self-record	12	-	-	28.75 ±4.5	Healthy	-	In-door	RGB	IDS UI-3160CP	1920X1080	120	-	-	Chest signal	Subject lying in supine position
**41**	[92]	HRV	Self-record	11	-	-	22±2	Healthy	Fitz scale: II to IV	In-door	RGB	Imaging source DFL-23UM021	-	115	-	40 cm	ECG, blood volume pulse sensor, skin	Rest, placing the right hand in cold water

																	conductance and respiration sensor	
**42**	[93]	HRV	Self-record	5	-	-	28.6± 2.3	Healthy	-	Indoor	RGB, NIR	NIR: Point Grey Grasshopper GS3-U3-23S6M-C; RGB: Point Grey Grasshopper GS3-U3-23S6C-C	1184X1200 ; 592X600	50	Red LED brick light, infra-red LED brick light	1.8 m	Philips IntelliVue MX700	Lying in a bed in supine position
**43**	[94]	HR, RR, BT	Self-record	5	-	-	-	-	-	Indoor	Thermal	Optrics PI-450 thermal imager	382X288	27	-	1.5 m	Pulse oximeter, manual counting, average value of the forehead region in thermal image	Sitting with movements (head up and down, forward and backward, turning right and left)
**44**	[95]	HR, HRV	Self-record	15	12	3	23-35	Healthy	Fair skin	Indoor	RGB	GoPro HERO3 silver edition, Logitech HD Pro Webcam C910	1280X720, 1920X1080	60; 15	Sunlight	0.3 m	Pulse oximeter	Sit still, lateral and forward/backward movements
**45**	[97]	HR	Self-record	3	-	-	-	Healthy	-	-	RGB	-	-	30	-	-	Pulse monitoring device	Normal rest, can talk to acquisition team
**46**	[98]	HR	Self-record	5	-	-	13-25 days	Infants	China	Indoor	RGB	Fluke TiX580	640X480	9	-	0.25-0.36 m	ECG	Infants lying in bed
**47**	[99]	HR, RR	Self-record	30	18	12	27.6-36.4 weeks	Preterm infants	White British, Asian, Black, mixed	Indoor	RGB	JAI 3-CCD AT-200CL digital video camera	1620X1236	20	Ambient light	-	Philips Intellivue MX800 patient monitor	Preterm infants lying in incubator bed
**48**	[100]	RR, BST	Self-record	26	-	-	-	ICU patients	-	Indoor	Thermal	Optris PI450i	382X288	4	-	-	Philips patient monitor	Lying in bed
**49**	[101]	HR, SpO_2	Self-record	28	-	-	0.6-4.7 days	Healthy infants	Fitz scale: I-VI	Indoor	RGB	Logitech C920	-	30	Ambient Fluorescent lamp	-	Pulse oximeter	Infant lying in bed
**50**	[102]	HR, RR	Self-record	10	-	-	2 to 95 days	Preterm infants	-	Indoor	RGB	Nikon D610, D5300	1920X1080	30	Ambient fluorescent light	1-2 m	Philips intellivue monitor	Infants lying in bed
**51**	[104]	HRV	Self-record	30	-	-	24-39	-	Light yellow skin, dark brown, white skin	Indoor and outdoor	RGB	Lenovo Phab Pro2	1920X1080	15	Varying illumination (150, 250, 380, 600, 1000 lux)	25-50 cm	ECG	Indoor: sitting in a chair with varying illumination and motion, Outdoor: passengers in a driving car, coffee shop
**52**	[105]	RR	Self-record	12	6	6	24±4	Healthy	-	Indoor	RGB	CCD RGB webcam	1280X720	30	Neon light, light through window	1.2 m	Different digital pressure sensor, SDP610 Sensirion	Sitting and breathing spontaneously
**53**	[106]	HR, RR	Self-record	9	9	0	Mean 23.6	Healthy	-	Indoor	RGB, Thermal	Imaging source DFK 23U618, FLIR A315,	640X480, 320X240	15	-	1 m	ECG, Respiratory belt	Standing, sitting with movement
**54**	[107]	HR	Self-record	40	24	16	18-60	-	-	Indoor	RGB	Logitech HD	640X480	30	Fluorescent lamp	50 cm	ECG	Static and dynamic
**55**	[108]	HR, RR	Self-record	9	-	-	GA: 25-40 weeks	Preterm infants	-	Indoor	RGB	Digital camera	1920X1080 or 1280X720	24 or 30	Sunlight through windows and fluorescent lamps	50 cm	Dräguer NICU monitor	Infants in the incubator bed

**56**	[109]	HR	Self-record	19	6	13	-	Infants	India	In-door	RGB, NIR, LWIR	NIR: Grasshopper 3 GS3-U3-23S6M-C; RGB: GS3-U3-23S6C-C; LWIR: Gobi-640-GigE, Vari-oCAM HD head 820S	RGB & NIR:1920X 1200, LWIR: 640X480, 1024X768	RGB & NIR: 25, LWIR: 25, 30	NIR LEDs, ambient lights, sunlight, radiant warmer	70 cm	Pulse oximeter	Infants lying in bed
**57**	[110]	HR	Self-record	10	5	5	20-35	Healthy	Fitz scale I-VI	In-door	RGB	Nikon AF-S	-	70	Florescent light	2 m	Pulse oximeter	Subject underwent two discrete episodes of hypoxia
**58**	[111]	HR, PTT	Self-record	12	6	6	20-37	Healthy	Fitz scale: I-VI	In-door	RGB	Logitech HD pro C920	640X480	30	Fluorescent light	40-60 cm	ECG, finger PPG	Sit still
**59**	[112]	HR	Self-record	15	10	5	20-30	-	Yellow skin	In-door	RGB	ELP-USB30W04MTR L21	640X480	30	Fluorescent lamp	2 m	Contact based HR monitor	Sit still or exercise gently
**60**	[113]	BP	Self-record	100	70	30	22-50	Healthy	Asian	In-door	RGB	CCD camera	640X480	20	Normal day light of 150 lux	60 cm	Electronic sphygmomanometer	Stay still
**61**	[114]	HR	Self-record	40	28	12	22-35	Healthy	China	In-door	RGB	Nikon CoolPix L610	600X800	30	Ambient light	-	Pulse oximeter	Sitting with slight movement
**62**	[115]	HR	Self-record	Lab:5 Home: 87, Panic attack: 9	-	-	Lab; 18-35, Home: 18-65, Panic: 19-34	-	-	In-door	RGB	iPhone	-	-	-	-	ECG, manual palpation	Before and after physical activity, panic attack
**63**	[116]	HR	Self-record	9	4	5	21-25	Healthy	-	In-door	RGB	HP laptop webcam Pavillion DM4/ Samsung Galaxy s4	Laptop: 340X480 / Smart phone: 1920X1080	30 / 30	Ambient light through a window, fluorescent lamp	1m	Biopac ECG, piezo resistive sensor	Stand still, lateral and frontal movements, breathing maneuvers (spontaneous breathing, Metronome breathing, Forced breathing)
**64**	[118]	IBI, HRV	Self-record	9	8	1	20s-60s	-	-	In-door	RGB	RGB-NIR sensor	640X480	300	KINOFLO lights, NIR lights	1.5 m	cPPG sensor	Relax, exercise-hand grip exercise, relax
**65**	[119]	BP	Self-record	4	4	0	22-25	Healthy	-	In-door	RGB	High speed camera MEMRECAM Q1m	1280X1024	500	Artificial solar lamp	-	Sphygmomanometer, ECG, PPG	High intensity (squat) exercise
**66**	[120]	HR, HRV	Self-record	25	17	8	18-34	Healthy	Fitz scale: I, II, III, IV	In-door	RGB	Scout scA640-120gc	658X492	120	Solux bulbs	1.5 m	Pulse oximeter, ECG	Stay still, head movements
**67**	[121]	HR	MoLi-ppg1	30	-	-	18-35	-	-	In-door	RGB	Logitech C920, Logitech C270, Canon LEGRIA HFG40	webcam: 800X600 or 1280X720; HD: 1920X1080	Webcam: 25, HD: 50	Fluorescent lamp	1 m	Contact PPG	Static, movements, recovery after exercise
			MoLi-ppg2	15	-	-	-	-	-		RGB	Webcam canyon 720p, HD video camera Panasonic	-	-	-	-	-	Static, speech, recovery after exercise
			UBFC-rPPG	42	-	-	-	-	-		RGB	Logitech C920HD Pro	640X480	30	Varying indoor light and sunlight	1 m	Pulse oximeter	Mathematical game
**68**	[122]	HR, BP	Self-record	15	-	-	20-38	Healthy	-	In-door	RGB	Google android tablet	1920X1080	15	Uniform white light	30-45 cm	Oscillometric BP and HR monitor, finger pulse sensor	Climbing stairs activity

**69**	[123]	BP	Self-record	85	55	30	61.6± 14.1	Healthy	Chinese	In-door	RGB	iPhone 6s	-	-	White LED	50 cm	Sphygmoma-nometer	Sit still
**70**	[124]	RR	Self-record	10	-	-	-	Healthy	-	In-door	RGB	Point Grey Flea 3 GigE	648X488	-	-	-	Polysomnog-raphy	Lying in bed
**71**	[125]	RR	Self-record	40	19	21	34.1± 12	-	-	In-door	Ther-mal	FLIR SC3000	320X240	30	-	120 cm	Subject finger flexion (up-ward and downward) in video	Subjects asked to breathe through a nose
**72**	[126]	BP	Self-record	8	7	1	22.4± 2.7	Healthy	-	In-door	RGB, ther-mal	iphone 7, Nip-pon TVS-200EX	RGB:1920 X1080, Thermal: 320X240	30,1	Bright (900-1000 lux)	50 cm, 60 cm	Finometer Model-2, Blood pressure cuff	Rest state with cold press test
**73**	[127]	BST	Self-record	50	Fe-ver: 9; Healt hy: 12	Fever: 16; Healt hy: 13	43+/-18 fe-ver; 32+/-19 healt hy,	Healthy, fever	-	In-door	Ther-mal	Thermopile ar-ray	48X47	6	-	30-50 cm	pulse sensor, 24-GHz micro-wave radar, Contact type clinical ther-mometer	Sit steady
**74**	[128]	RR	Self-record	107	42	59	58.5± 15.8	Post-sur-gical pa-tients	-	In-door	FIR	FLIR T450sc	-	30	-	1 m	GE healthcare patient moni-tor, Visual in-spection	Lying in bed
**75**	[129]	HR	Self-record	2	-	-	-	-	-	In-door	RGB	-	-	30	Natural light	-	Polar H10	Exercise on treadmill
**76**	[130]	HR	Self-record	25	18	7	20-28	-	-	In-door	RGB	Surface 4 tablet	1920X1080	29.97	Dynamic light - 150 lux, 300 lux light	-	Polar H7 mon-itor	stationary, rotation action (roll), mixed action (yaw, pitch, scaling and translation) and 4 ft walk to-wards the camera
**77**	[131]	HR, RR, BT	Self-record	22 healthy; 41 influ-enzas	-	-	23.4 healt hy, 45.0 influ-enza	Healthy, influenza	-	In-door	RGB, ther-mal	RGB camera DFK23U618, FLIR A315	RGB: 640X480; Thermal: 320X240	15	-	1 m	ECG, Pulse ox-imeter, Respir-atory effort belt, clinical thermometer	Standing
**78**	[132]	HR, BST	Self-record	HR: 7; BST: 4	-	-	-	-	-	In-door	RGB, ther-mal	Logic C920, FLIR Lepton thermal camera	-	-	-	30-60 cm	Thermometer, Polar H7 HR band	Sitting and slightly talking or movement
**79**	[151]	HR	Self-record	15	8	7	30.6± 3.7	Healthy	-	In-door	RGB	Webcam	640X480	30	-	-	ECG	Computer based psychological test, doctor game, two interactive web calls
**80**	[152]	RR	Self-record	28	4	24	Inter-quar-tile range 51-77	Post-An-esthesia Care Unit Pa-tients	-	In-door	LWIR	Infratec Vario-CAM HD head	1024X768	30	-	-	Philips Intel-liVue MP30 monitor	Lying in bed
**81**	[153]	HR	Self-record	10	-	-	-	-	-	Out-door	RGB	Go-Pro HERO 3+	1920X1080	30	-	-	Transmissive pulse oximeter	Driving
**82**	[154]	HR	Self-record	6	3	3	-	Healthy	-	In-door	RGB	Chicony USB 2.0 camera webcam	640X480	29.97	Fluorescent lamps and natural light	35-60 cm	Pulse oximeter	head to camera, head side to cam-era
**83**	[156]	HR	Self-record	100	90	10	20-55	Healthy	-	In-door, Out-door	RGB, NIR	Kinect 2.0	RGB: 1920*1080; NIR: 512*424	15	Dark environ-ment with NIR light (0-80 lux)	60 cm (40-80 cm)	Finger pulse oximeter	Stay still, Strenuous exercise, mul-tiple subjects, driving
**84**	[157]	HR	MAHNOB-HCI	27	12	15	19-40	-	-	In-door	RGB	Allied Vision Sting-ray F-046C	780X580	61	-	40 cm	ECG	Emotion elicitation and implicit tagging

			Self-record	19	-	-	20s-30s	-	Middle east Asia and Asia	Outdoor	RGB	GoPro HERO3+	1920X1080	30	-	-	Contact based pulse sensor	Driving condition
**85**	[158]	HR	Self-record	10	-	-	-	-	-	Indoor	RGB	Microsoft Kinect 2.0	1920X1080	21	-	0.5-2.5 m	Pulse oximeter	varying distance with different angle
**86**	[159]	HR	Self-record	4	-	-	-	-	-	Indoor	RGB	Macbook air	480p	30	-	-	PPG signals at the pointing finger	Remain still
**87**	[160]	HR	Self-record	9	-	-	-	-	-	Indoor	RGB	Intel RealSense D435	640X480	30	Working space light	-	SpO_2 sensor	Sitting posture with slight movement
**88**	[163]	HR	OBF	100	61	39	31.6± 8.8	Healthy	Caucasian Asian, others	Indoor	RGB	Blackmagic URFA mini	1920X2080	60	LED light	1 m	ECG	Resting state, post exercise state
			MAHNOB-HCI	27	12	15	19-40	-	-	Indoor	RGB	Allied Vision Sting-ray F-046C	780X580	61	-	40 cm	ECG	Facial expression and voluntary head motion
**89**	[164]	HRV	Self-record	16	9	7	-	-	Light, dark skin	Indoor	RGB	Blackfly BFLY-U3-23S6C	-	30	Ambient, LED arrays, laptop screen light	-	Pulse oximeter	stay still, reading, watching, talking, deep breath
**90**	[165]	HR	Self-record	11	7	4	22-32	Healthy	-	Indoor, outdoor	RGB	FLIR BFLY-U3-23S6C-C	960*600	30	ambient and fluorescent light	1.5 m	Finger blood volume pulse sensor	Sitting, Standing, Running(treadmill)
**91**	[166]	RR	Self-record	20	16	4	20-38	-	-	Indoor	RGB, thermal	Thermal imager MAG62, RGB camera	RGB: 640X480	10	-	-	Sleep respirator monitor	Sitting with movements
**92**	[167]	HR	Self-record	8	-	-	-	-	-	Indoor	RGB	Logitech c270	-	-	-	-	Pulse oximeter	Sit quietly, shake their heads normally, daily interactions (e.g., talk, smile)
**93**	[172]	BP	Self-record	1328	-	-	18-87	Arrhythmia, Diabetes, Hypertension, Lung disease	East asian, white, south asian, middle eastern, black, hispanic/latino, mixed/unknown	Indoor	RGB	iPhone 6 plus	720p	30	LED	40-60 cm	CNAP Monitor 500	Sitting comfortably
**94**	[173]	HR	Self-record	1	1	0	-	Healthy	Asian	Indoor	RGB	18-bit camera (Xviii: VIEW-Plus)	-	30	Varying (5-300 lux)	1 m	ECG polygraph	Face was fixed using chin rest
**95**	[178]	HR	Self-record	1	1	0	-	-	-	Indoor	RGB	-	-	25	-	80 cm	Finger pulse oximeter	Sit still
**96**	[182]	HR	Self-record	-	-	-	-	-	-	-	RGB	Logitech C922	640X480	30	-	-	Pulse oximeter	-
**97**	[184]	RR	Self-record	30	30	0	-	Healthy	-	Indoor	RGB	IDS uEye-2220	-	20	Regular office light	1.5 m	Capnography, visual inspection	Sitting quietly with varying respiratory frequency and cloth
**98**	[185]	RR	Self-record	9	5	4	25-32	-	-	Indoor	RGB	Galaxy S9+	1920X1080	240	Stable amount of light	0.6 m	Manual counting by observing the torso	Lying in bed on the side and regular breathing
**99**	[187]	HR	Self-record	11	-	-	22-24	-	-	Indoor	RGB & NIR	JAI AD-130GE	1296X966	30	Varying illumination (0.4 lux, 1 lux, 600 lux)	-	Pulse oximeter	Sit still
**100**	[188]	HR	Self-record	5	-	-	-	-	-	Indoor	RGB	Kinect 2 somatosensory camera	-	15	Strong, natural, dark light	0.5,1,2m	Pulse touching	Standing

**101**	[189]	HR	Self-record	11	5	6	-	-	-	In-door	RGB	webcam	1280X720	15	Different lighting	30-40 cm	Pulse oximeter	Rest and different activities
**102**	[190]	HR	Self-record	9	-	-	-	-	-	In-door	RGB	iPhone 6s	-	-	Fluorescence light and day-light	-	ECG- based chest belt Polar H7	Treadmill exercise or elliptical machine exercise
**103**	[191]	HR	Self-record	30	-	-	-	-	-	In-door	RGB	Webcam	640X480	30	Ambient light	0.5 m	ECG Biocare ECG-2000	Sitting with illumination variations
**104**	[197]	HR	Self-record	11	9	2	30±10	-	Caucasian, black african, asian	In-door	RGB	Dell XPS 15 laptop	720p	30	Ambient light	-	wrist worn contact PPG sensor	Stationary and varying illumination
